# 2388 Utilizing a reviewer database to facilitate integration of an investigator-focused translational research and career development program across the state of Indiana

**DOI:** 10.1017/cts.2018.233

**Published:** 2018-11-21

**Authors:** R. L. Coffee, Julie Driscol, Tammy J. Saydyk, Anantha Shekhar, Scott C. Denne, Joe D. Hunt

**Affiliations:** Indiana University School of Medicine & Indiana CTSI

## Abstract

OBJECTIVES/SPECIFIC AIMS: The Indiana CTSI is investigating innovative approaches to integrate resources that will enrich scientific investigators. Our goals are to enhance the availability and communication among CTSI resources, for example internal funding, and to expand existing mentorship. METHODS/STUDY POPULATION: Developed a reviewer database that serves to streamline reviewer identification, decrease reviewer fatigue, and promote collaboration among disciplines. We started with a pool of NIH-funded investigators from across the Indiana CTSI core institutions and merged this list with previous CTSI reviewers and internal funding awardees. To expand this list, names and expertise from new faculty hires were added. RESULTS/ANTICIPATED RESULTS: Though this tool is relatively new, we have already observed an increase in junior faculty awareness and engagement with the CTSI. This database allows for increased opportunities of junior faculty to serve as reviewers and to refine grant writing skills and provides a platform for networking and collaborating across disciplines. It also allows for increased integration of programs with a shared reviewer database and promotes grant review standardization. DISCUSSION/SIGNIFICANCE OF IMPACT: Our database utilization seeks to decrease the time for junior faculty to obtain their first extramural grant, to enhance promotion and tenure packages, strengthen integration among CTSI programs, increase interactions between clinical and basic science investigators, and promote team science.

